# Heart Transplantation in Congenital Heart Disease: In Whom to Consider and When?

**DOI:** 10.1155/2013/376027

**Published:** 2013-02-07

**Authors:** Christine H. Attenhofer Jost, Dörthe Schmidt, Michael Huebler, Christian Balmer, Georg Noll, Rosmarie Caduff, Matthias Greutmann

**Affiliations:** ^1^Division of Cardiac Surgery, University Hospital Zurich, Zurich, Switzerland; ^2^Division of Cardiology, Department of Paediatric Cardiology and Congenital Heart Disease, University Children's Hospital Zurich, 8032 Zurich, Switzerland; ^3^Department of Pathology, University Hospital Zurich, Zurich, Switzerland; ^4^Congenital Heart Disease, Division of Cardiology, University Hospital Zurich, Raemistrße 100, 8091 Zurich, Switzerland

## Abstract

Due to impressive improvements in surgical repair options, even patients with complex congenital heart disease (CHD) may survive into adulthood and have a high risk of end-stage heart failure. Thus, the number of patients with CHD needing heart transplantation (HTx) has been increasing in the last decades. This paper summarizes the changing etiology of causes of death in heart failure in CHD. The main reasons, contraindications, and risks of heart transplantation in CHD are discussed and underlined with three case vignettes. Compared to HTx in acquired heart disease, HTx in CHD has an increased risk of perioperative death and rejection. However, outcome of HTx for complex CHD has improved over the past 20 years. Additionally, mechanical support options might decrease the waiting list mortality in the future. The number of patients needing heart-lung transplantation (especially for Eisenmenger's syndrome) has decreased in the last years. Lung transplantation with intracardiac repair of a cardiac defect is another possibility especially for patients with interatrial shunts. Overall, HTx will remain an important treatment option for CHD in the near future.

## 1. Introduction

Since the first successful heart transplantation in a human by Christiaan Barnard in 1967 [1], heart transplantation (HTx) has evolved from its experimental stage to an established treatment option for patients in end-stage heart failure [1–3]. In 1967, the allograft survived six hours. Only with the introduction of cyclosporine, further pediatric transplants followed in the 1980's. While historically most patients undergoing orthotopic HTx suffered from end-stage ischemic or dilated cardiomyopathy, the proportion of patients with congenital heart disease has increased [4].

Congenital heart disease (CHD) is common affecting 0.4–1% of the population. With the advent of modern heart surgery, the majority of these patients, even those with complex lesions, now survive to adulthood [5, 6]. Thus, there is an increasing number of adult survivors with complex CHD [5]. Despite these incredible improvements in surgical repair options and thus outcome, many survivors of infant heart surgery for CHD are not cured and some remain at high risk of developing end-stage heart failure as young adults. Heart failure contributes importantly to the late morbidity and mortality in adult CHD [7–9]. In the end-stage of their disease, orthotopic HTx currently often remains the only viable treatment option. Given these changes in epidemiology of patients with CHD, over the next few decades we expect a rapidly increasing population of adults with CHD that will be assessed for HTx [10].

In the paper we will discuss specific issues and challenges in the pre, peri, and post-transplantation period of these patients and illustrate some of these pertinent issues with real-life patient vignettes.

## 2. Changing Mortality Patterns and Causes of Death in CHD

To understand current and to predict future patient cohorts at risk for end-stage CHD, it is important to review the changes and evolution of surgical treatment strategies for complex CHD over the last few decades. This evolution will have a major impact on the total number of individuals with certain complex congenital heart lesions and the age distribution within these cohorts. It is also important to analyze causes and modes of deaths and their changes.

Recent studies have demonstrated that childhood mortality in CHD has almost disappeared and mortality has shifted almost entirely to adulthood [11]. The main reasons for HTx are shown in Table 1. While primary transplantation for newborns and infants with hypoplastic left heart syndrome or the severest forms of Ebstein's anomaly of the tricuspid valve may be valuable treatment options in selected cases, surgical palliation along the Norwood route is nowadays attempted at most centers. The underlying congenital cardiac defects in 164 adults with CHD who died from their disease during followup in a single center reported by Oechslin et al. were shunt lesions with Eisenmenger physiology (29%), patients with repaired tetralogy of Fallot (10%), patients with univentricular hearts (8%), and patients with systemic right ventricles (6%) [7]. In a national registry for ACHD patients in the Netherlands including 6,933 patients, 197 patients died at a median age of 49 years: the most common diagnoses were atrial septal defects (17%), tetralogy of Fallot (12%), aortic stenosis/bicuspid aortic valves (9%), systemic right ventricles (9%), and univentricular hearts (6%) [12]. Within these series, end-stage heart failure contributed substantially to late mortality and contributed to roughly one third of all late deaths [7–9]. The specific etiology of heart failure in CHD is summarized in Table 2.

## 3. Congenital Cardiac Defects at Risk for End-Stage Heart Failure

While there has been a slow decline in the number of adult heart transplants for acquired heart disease over the last few decades, the proportion of HTx for end-stage ACHD has slightly decreased [14]. The proportion of CHD among transplant recipients is also very much dependent on the age at transplantation. According to the 2010 report of the International Society for Heart and Lung Transplantation Registry CHD was present in 63% of patients undergoing HTx < 1 year of age, 37% of those 1–10 years of age, 25% in patients 11–17 years of age, and only 2% of adult recipients. [15]. Given the expected changes of the epidemiology of CHD, these trends are expected to further change in the near future with a shift of transplants from the pediatric to the adult area of congenital heart disease. Thus, the face of heart transplantation is slowly changing [2, 4].

In single center studies, the most frequent individual congenital cardiac diagnoses in patients undergoing HTx are single ventricle physiology with or without Fontan palliation, transposition complexes with systemic right ventricles (complete transposition after atrial switch procedures or congenitally corrected transposition of the great arteries), and patients with repaired or palliated tetralogy of Fallot [16].

## 4. Etiology of Heart Failure in ACHD

Although neurohumoral activation in CHD patients with circulatory failure is very similar to patients with acquired heart disease, causes of circulatory failure in CHD are more diverse [17]. Etiology of heart failure (see Table 2) includes primary systolic or diastolic ventricular dysfunction and/or progressive valvular regurgitation or stenosis. Factors predisposing to ventricular myocardial dysfunction are long-standing ventricular volume or pressure overload due to valvular regurgitation, congenital shunt lesions, or surgically created systemic to pulmonary shunts. Additional contributing factors are long-standing cyanosis, prior surgical ventricular incision, and uncontrolled arrhythmias. Adverse ventriculoventricular interactions are increasingly recognized as an important contributor to systemic ventricular dysfunction in patients with dilatation and dysfunction of the subpulmonic ventricle [18]. Additionally, there may be inherited abnormalities of the myocardial structure in patients with CHD such as noncompaction which is more common in Ebstein's anomaly [19] and other patients with CHD [20]. In patients with a systemic right ventricle such as patients with congenitally corrected transposition of the great arteries (CCTGA) and patients with complete transposition of the great arteries (D-TGA) repaired with an atrial switch operation (Mustard or Senning procedure), an oxygen supply-demand mismatch may lead to myocardial ischemia and fibrosis. In these patients substantial atrioventricular valve regurgitation is often present and associated with an increased preload and is felt to be an ominous sign of impending ventricular failure [21–23]. Both systolic dysfunction of the systemic RV and progressive tricuspid regurgitation have been associated with increased mortality.

In CCTGA retrospective multi-institutional studies clearly demonstrate an increasing incidence of systemic and ventricular dysfunction and clinical congestive heart failure with advancing age [24, 25]. Even in patients with CCTGA and no significant associated lesions, more than one third had congestive heart failure by the fifth decade of life [24, 25]. As clinical worsening is often caused by progressive systemic AV valve regurgitation, timely systemic atrioventricular valve replacement may offer the best prevention of further deterioration and may postpone or even prevent the need for HTx.

The Fontan circulation for palliation of patients with univentricular physiology represents a unique form of modified CHD. Most adult survivors with this type of physiology still have modified atriopulmonary types of Fontan procedures. In the future, novel cohorts of patients with lateral tunnel Fontan's or total extracardiac cavopulmonary anastomosis will emerge. The rate of complications among Fontan patients increases in the third and fourth decade of life. Given the evolution of these procedures over the last 4 decades these cohorts are still, emerging and in the future we expect many more patients with “failing Fontans.” The malfunction of the Fontan circulation has many contributors, with single ventricular dysfunction being just one component. In addition, in this patient group atrial arrhythmias are very common, and chronically elevated systemic venous pressures often lead to hepatic fibrosis or cirrhosis. Protein losing enteropathy is a dreaded long-term complication and associated with poor long-term survival. While surgical conversion of an atriopulmonary Fontan to a total cavo-pulmonary anastomosis may be a good treatment option for selected patients, many with advanced circulatory failure and multiorgan affection may have a very high perioperative mortality, and thus transplantation may be a more favorable treatment option in these individuals (see patient vignette 1).

## 5. Case Vignette 1: A 31-Year-Old Patient with Failing Fontan

**Figure 1:**
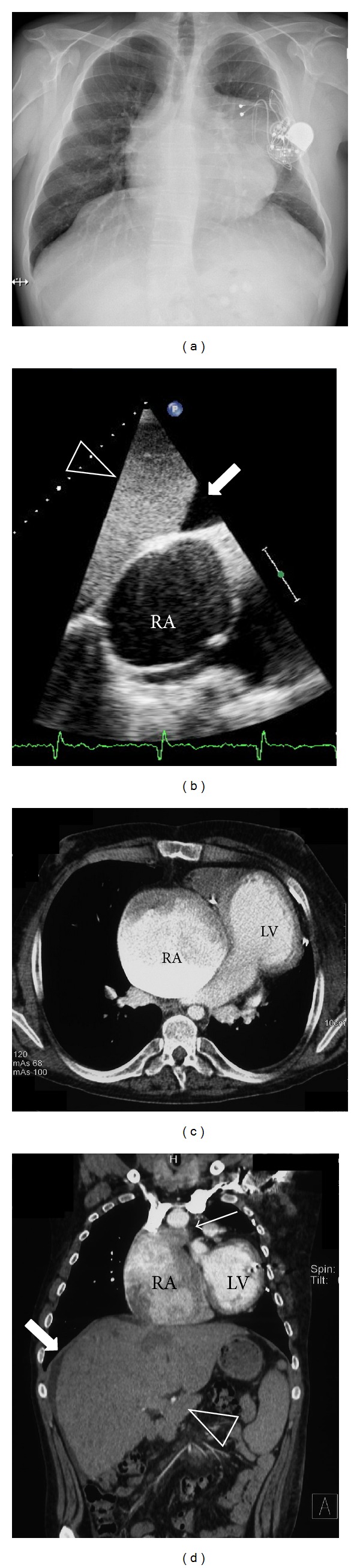
31-year old patient with tricuspid atresia, atrial and ventricular septal defects, and severe infundibular pulmonary stenosis. He underwent surgical palliation with a Fontan procedure, initially using an atrial to right ventricular conduit, later converted to an atriopulmonary Fontan-Kreutzer-Anastomosis due to conduit failure. After increasing heart failure and 5 cardiac surgeries, he underwent heart transplantation. (a) Chest X-ray with massive cardiac enlargement and the epicardial pacemaker. (b) Subcostal echocardiographic view showing severely dilated right atrium (RA) with spontaneous echocontrast and perihepatic ascites (bold arrow). (c) Axial plane of chest-computed tomogram showing giant right atrium (RA) and normal-sized left ventricle (LV). (d) Coronal plane of chest and abdominal computed tomography showing enlarged right atrium (RA) with connection to the main pulmonary artery (arrow) and hepatomegaly (arrow head).

**Figure 2:**
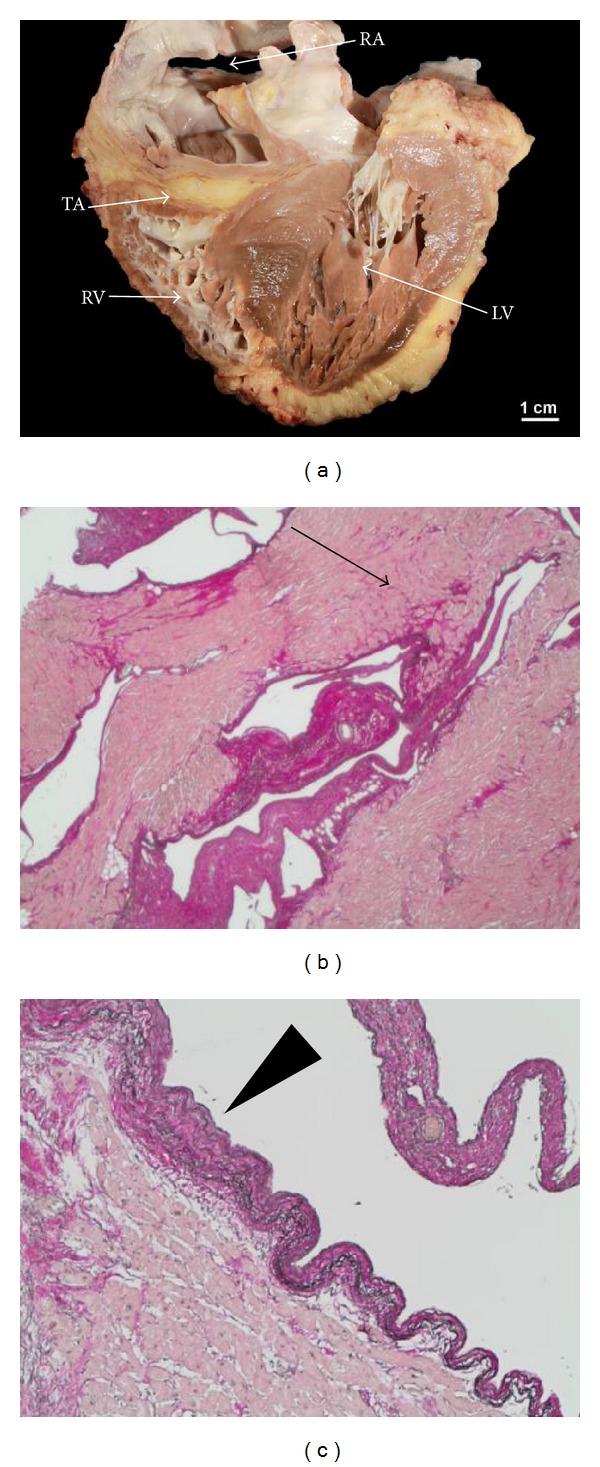
Explanted heart of a 31-year-old patient with failing Fontan. (a) Macroscopic view of the explanted heart, showing the tricuspid atresia (TA) with hypoplastic right ventricle (RV), grossly dilated right atrium (RA) and left ventricle with septal hypertrophy. (b) and (c) show histologic cuts of the right ventricular myocardium with areas of interstitial myocardial fibrosis (arrow) and areas of marked endomyocardial fibroelastosis (arrow head).

The patient was born with tricuspid atresia, atrial and ventricular septal defects, and severe infundibulary pulmonary stenosis. He underwent surgical palliation with a Fontan procedure, initially using an atrial to right ventricular conduit, later converted to an atriopulmonary Fontan-Kreutzer-Anastomosis due to conduit failure. Until the age of 11 years he already had had a total of 5 sternotomies. At the age of 17 years he needed implantation of an epicardial pacemaker for sinus node dysfunction. In his late 20s he experienced progressive decline of exercise capacity developed refractory atrial arrhythmias and finally progressive Fontan-failure with cardiac cachexia, intractable ascites, liver cirrhosis, mild protein losing enteropathy, and severe renal dysfunction (see Figure 1). He had recurrent hospital admissions for heart failure and worsening renal function. At the age of 31 years, he was listed for HTx and received an organ after 9 months on the waiting list. At the time of transplantation extensive reconstruction of the pulmonary arteries had to be performed. He had a surprisingly smooth postoperative course with immediate recovery of renal function and was discharged 4 weeks after transplantation. Macroscopic and histologic images of the explanted heart are shown in Figure 2.

As survivors of Norwood palliation are only just reaching adulthood, we are not dealing with this entity in this paper. Cardiac transplantation in children with visceral heterotaxy as described by Larsen et al. can be an option with a 10-year mortality of 50% [26]. In case vignette 2, we describe one of our patients with visceral heterotaxy which is not considered for HTx yet.

## 6. Case Vignette 2: Complex Cardiac Anatomy

**Figure 3:**
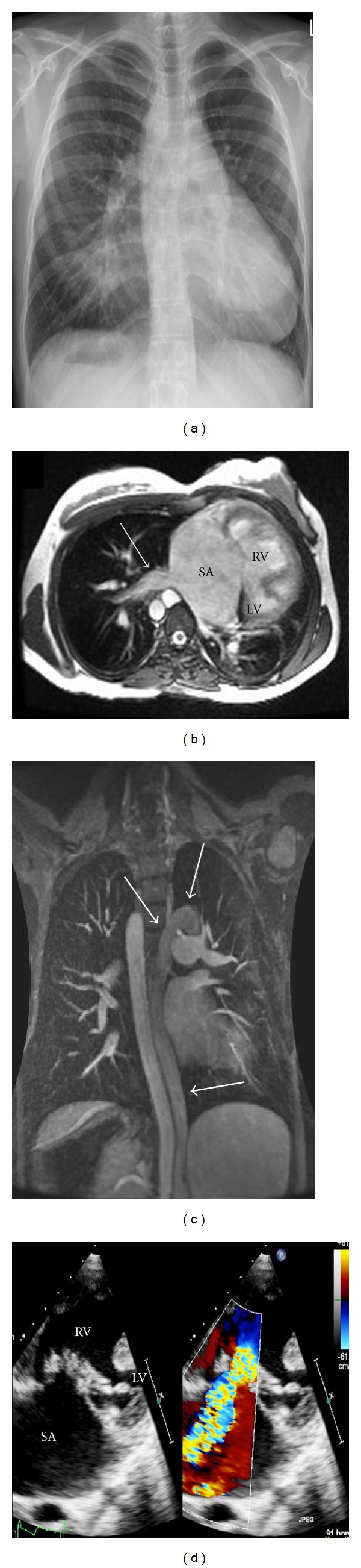
24 year old patient with heart failure and complex cardiac anatomy in the setting of left atrial isomerism. (a): Chest X-ray. (b): Axial slice of cardiac magnetic resonance imaging, showing a single atrium (SA) with direct connection of hepatic veins (arrow), dominant right ventricle (RV) and hypoplastic left ventricle (LV). (c): Coronal slice of cardiac magnetic resonance imaging showing hemiazygous continuation to left superior caval vein (arrows) in the absence of a right superior caval vein. (d): Apical echocardiographic view demonstrating the dominant right ventricle (RV), the hypoplastic left ventricle (LV), the enlarged single atrium (SA) and the dysplastic tricuspid valve. On the right hand of the panel color Doppler demonstrates severe tricuspid regurgitation.

A 24-year-old patient was assessed for cardiac transplantation. She was born with left atrial isomerism with venous drainage of the lower body half via hemiazygos continuation to a single left superior caval vein. Liver veins drain directly to a single atrium. Intracardiac defects comprised a single atrioventricular valve with a severely hypoplastic ventricle and severe subvalvar pulmonary stenosis (see Figure 3). She underwent palliation with right modified Blalock-Taussig-Shunt. At the age of 18 years she underwent emergent recanalization and stenting due to thrombotic occlusion of the Blalock-Tausig-Shunt. At that time slightly elevated pulmonary artery pressures were recorded, precluding a Fontan-procedure. In her early twenties the patient developed severe atrioventricular valve regurgitation with progressive exercise intolerance. Atrioventricular valve repair was discussed but was declined due to the expected very high perioperative mortality. Transplantation was deemed technically possible, though challenging given the complex venous anatomy and borderline elevated pulmonary pressures. Prior to listing, she is currently kept under close clinical surveillance with serial imaging and cardiopulmonary exercise testing with the aim to list the patient for transplantation in the event of further deterioration of exercise capacity.

## 7. Specific Considerations for Heart Transplantations in Congenital Heart Disease

In patients with ACHD and end-stage circulatory failure, the following three issues add to the complexity of transplantation: (1) tools for prognostication in ACHD patients are not well established and may differ between different congenital cardiac disease entities. Whether or not it is legitimate to adopt prognostic scores from patients with acquired heart disease remains to be seen. As complicating factors, some of the patients with CHD have anomalies of the systemic or pulmonary venous return or situs inversus or dextrocardia. They may additionally need concomitant aortic arch correction in patients with hypoplastic left heart syndrome needing HTx. Also assessment of pulmonary vascular resistance after previous Glenn and Fontan circulation may be difficult. (2) Most adults with CHD had previous open heart surgery necessitating often multiple interventions, which adds to the complexity of the surgical procedure at the time of transplant. Additionally, aortopulmonary collaterals increase the bleeding risk. In the presence of increased pulmonary venous return, the operating field may be hard to visualize sufficiently. In some patients, perfusion with the heart-lung machine may be almost impossible necessitating circulatory arrest in deep hypothermia. (3) Because of the previous use of blood transfusions or repair with homograft tissue, the rate of preformed HLA antibodies is increased.

To address these difficulties, a close collaboration among ACHD specialists, heart failure specialists, transplant surgeons and anesthesiologists is needed for optimal care of these patients. 

## 8. Prognostication of Survival

Timely identification of possible candidates for HTx is essential for patients without but also with CHD. In patients with acquired heart disease, maximal oxygen consumption per unit time (*V*_O_2__) has been used as an important criterion for listing for transplantation. Traditionally *V*_O_2__ maximum of <12–14 mL/kg/min in patients without beta-blocker treatment and <10 mL/kg/min in those on beta blocker therapy have been used as one of the criteria for listing for transplantation. Although it has been shown in a study with ACHD patients that decreased *V*_O_2__ is associated with an increased risk of hospital admission or death [27], it is not known whether the same *V*_O_2__-thresholds should be used for listing for transplantation and whether thresholds are independent of the underlying congenital cardiac defect. In a large study 1,375 consecutive adult patients with CHD (age, 33 ± 13 years) were included who underwent cardiopulmonary exercise testing at a single center over a period of 10 years with a median followup of 5.8 years. In this group, 117 patients died with the risk of death increased with lower peak *V*_O_2__ and lower heart rate reserve [28]. Of note, self-reported exercise tolerance poorly correlated with objective measures of exercise capacity and thus serial standardized exercise testing is strongly recommended in long-term followup [29]. So far, BNP levels have been shown to be predictors of sudden cardiac arrest (SCA) and ventricular arrhythmias in patients without CHD [30]. BNP levels also correlate with diminished function of the systemic right ventricle and parallel the degree of tricuspid regurgitation and thus outcome [31]. Recently, in a study involving 181 patients with Eisenmenger syndrome (31% with Down syndrome) during a median followup period of 3.3 years, 20 patients (7 with Down syndrome) died [32]. Higher BNP concentrations were predictive of all-cause mortality on univariate analysis in patients with or without Down syndrome. On multivariable Cox-proportional hazard analysis, BNP predicted survival independently of renal function, Down syndrome, or 6 min walk test distance (*P* = 0.004). Temporal increases in BNP concentration were also found to predict mortality [32].

As for acquired heart disease anemia, hyponatremia, and renal dysfunction [33–35] have been identified as markers for mortality risk in ACHD patients. Further additional risk factors such as pulmonary hypertension, recurrent hospital admissions for heart failure or cardiac cachexia may be important factors for adverse outcome. Even more important as the result of single testing may be changes during followup. For optimal long-term assessment of patients with ACHD we thus recommend to use standardized serial followup examinations following strict institutional protocols. In the absence of systematic prospective validation of risk factors decision making about timing of transplantation in ACHD patients has to be individualized. For this purpose all available individual risk factors and modifiers have to be taken into account and weighed against the expected individual perioperative morbidity and mortality of a transplant procedure. The risk factors for HTx are summarized in Table 4.

Many adults with congenital heart diesease are at inreased risk of SCA. In individual patients risk stratification is however difficult and depends on the type of underlying defects and additional factors. Prospective studies demonstrating a benefit for primary prophylaxis of AICD-implantation are unfortunately lacking, and risk stratification depends on retrospective analysis of single or multi center cohorts. In a recent single center study, 936 adults with previously repaired CHD and a mean age at first examination of 21 ± 7 years were followed during 9 ± 7 years. During followup, SCA occurred in 22 patients (2.6 per 1,000 person-years) with highest incidence in patients with transposition complexes [36]. Age at initial examination and severely impaired subaortic ventricular systolic function were independent risk factors for SCA (severe subaortic ventricular systolic dysfunction, adjusted hazard ratio 29, 95% confidence interval 11 to 72, *P* < 0.001). SCA occurred in 23% of patients with severe subaortic ventricular systolic dysfunction versus 0.7% of patients with nonsevere decreased subaortic ventricular function (*P* < 0.001). In contrast, a multicenter study of patients with AICD implantation for the transposition of the great arteries after atrial switch procedures did not show a benefit in those implanted for primary prevention and a high rate of lead complications and inappropriate shocks [37]. Thus, currently, the decisions for implantation of AICDs in patients on the waiting list for HTx must be individualized. Multicenter registries would be helpful to define benefit in the future.

## 9. Case Vignette 3: A Patient with Failing Senning and Sudden Cardiac Death While Awaiting Transplantation

**Figure 4:**
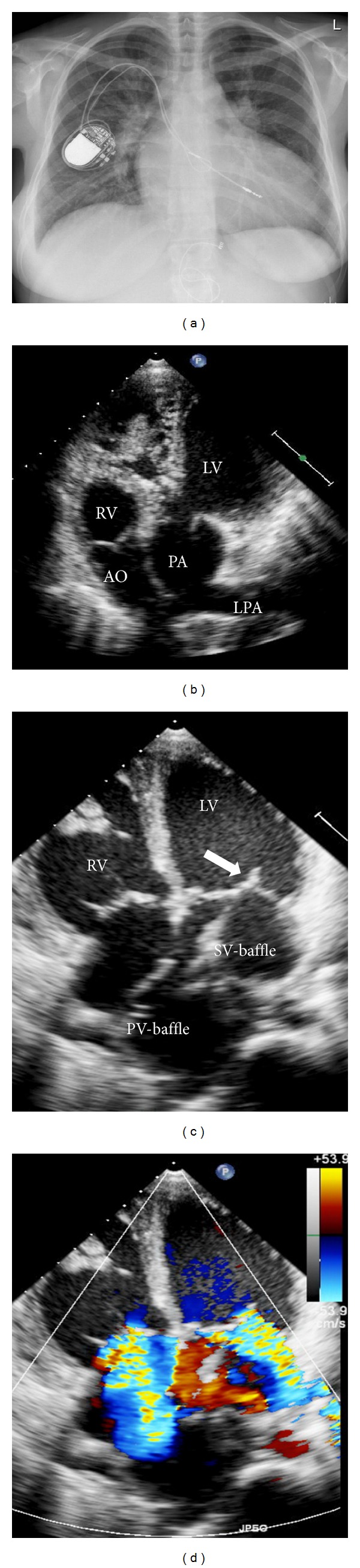
32-year-old patient with severe biventricular dysfunction after atrial switch operation for complete transposition of the great arteries with pacemaker leads in the subpulmonic left ventricle and the systemic venous baffle. (a) Chest X-ray with transvenous pacemaker leads. (b) Apical “5-chamber”-view showing the aorta arising from the morphologic right and the pulmonary artery from the subaortic left ventricle (RV: right ventricle, LV: left ventricle, AO: aorta, PA: main pulmonary artery, LPA: left pulmonary artery). (c) Apical 4-chamber view showing the pulmonary venous and the systemic venous baffles, redirecting venous blood. The arrow points to the pacemaker lead in the subpulmonic left ventricle. (d) Apical 4-chamber view with colour Doppler demonstrating severe (subpulmonic) mitral and severe (systemic) tricuspid regurgitation.

A 32-year-old woman, born with complete transposition of the great arteries and ventricular septal defect, was referred for transplant assessment. She had an atrial switch operation (Senning technique) at the age of 7 months. Surgical repair was complicated by complete heart block with the need for implantation of a pacemaker. At age 32, she developed biventricular heart failure with severe pulmonary hypertension. Exercise capacity was severely decreased (peak oxygen consumption 8 mL/kg/min). Echocardiography showed biventricular dilatation, severe biventricular systolic dysfunction, and severe regurgitation of both atrioventricular valves (see Figure 4). Cardiac catheterization demonstrated borderline pulmonary vascular resistance (3.3 WU). The patient was listed for cardiac transplantation. Subsequently she had several admissions for decompensated heart failure. She died 3.5 months after listening due to sudden death at home. Prior to sudden cardiac death she had never experienced syncope and was never documented to have ventricular arrhythmias.

## 10. Contraindications for Heart Transplantation in ACHD

Absolute and relative contraindications according to the ACC/AHA guidelines for the management of ACHD are summarized in Table 3. 

In ACHD patients with renal dysfunction in the pretransplant period related to cardiac dysfunction but without obvious renal disease, renal function often improves after transplantation (see case vignette 1). Most ACHD patients considered candidates for HTx are still young, and thus, atherosclerotic disease as a risk factor for adverse outcome in this population is rare. In ACHD patients, particular attention has to be paid on pulmonary vascular resistance (PVR). A fixed PVR index of >5 WU/m^2^ or a transpulmonary gradient >15 mmHg not responsive to vasodilator therapy are deemed to be contraindications. Of note, in patients with Fontan palliation determination of PVR can be difficult in the presence of venovenous collaterals and intracardiac shunts.

Patients with ACHD and irreversible pulmonary hypertension may be candidates for combined heart-transplantation or lung transplantation with surgical repair of the underlying congenital heart lesion at the time of transplantation [38]. The latter approach is usually reserved for patients with relatively simple defects such as simple shunt lesions. 

In the evaluation of patients for transplantation standardized checklist such as the one proposed by the Toronto ACHD Heart Transplant Evaluation team are helpful [10]. 

Complex cardiac anatomy (i.e., heterotaxy syndromes), multiple previous sternotomies or extensive collateral vessels may be relative contraindications for HTx. Early involvement of transplant surgeons and congenital cardiac surgeons is of paramount importance to address these difficulties and to assess transplant eligibility of an individual patient. Once such a patient is listed, availability of surgeons with the required expertise at the time of transplantation has to be ascertained. Patients with situs inversus are the most difficult anatomy for HTx. In patients with prior Glenn or Fontan surgery, the pulmonary artery has to be reconstructed with excess donor tissue. The same is true for the aortic arch in hypoplastic left heart syndrome. 

## 11. Waiting List Mortality in ACHD

Patients with ACHD often are identified at a relatively early stage and thus are usually listed at a low urgency status and consequently spend more time on the waiting list [39]; there is a conflict of search for the ideal donor and transplant environment versus the increased waiting list mortality seen in ACHD [40]. 

Important is the recognition that ACHD patients do receive ICDs less often than patients with acquired heart disease [40]. Although general predictors for the benefit of primary prevention of AICDs in ACHD cohorts are lacking, the recognition that sudden cardiac death contributes substantially to waiting list mortality in these patients suggests that a more liberal use of AICDs in ACHD patients listed for HTx may reduce waiting list mortality [41].

As mentioned by Burchill and Ross in their review [10], clinicians must carefully balance risks of premature listing against progressive heart failure and increased waiting list mortality. This is one of the most difficult tasks we encounter as cardiologists caring for patients with complex, end-stage CHD. 

Increasingly, mechanical circulatory support is used in patients with CHD prior to HTx in up to one third of children [42] without an impact on long-term mortality. In adults, mechanical circulatory assistance does not improve waiting list probably due to a combination of complex reoperative surgery and poor preoperative health [14]. Mechanical circulatory support of the single ventricle circulation is sometimes the only option for saving the patient's life and poses mechanical and physiological challenges. When late failure occurs, beside ECMO support, ventricular assist device (VAD) implantation may be considered as a bridge to transplantation. In vitro tests have evaluated different VADs especially for this setting [43–45]. Even if clinical experiences with long-term VAD support are still limited due to the complexity of the patients, the successful use of the Berlin Heart Excor up to 363 days has been published in case reports or case series [46], both in a “univentricular” [47] and a “biventricular” setting [48]. Small implantable devices like the Heartware HVAD may offer new options for these patients as reported by the Berlin group [49].

## 12. Outcome after Heart Transplantation for Congenital Heart Disease

Compared to patients with acquired heart disease, those with ACHD face an increased risk of perioperative death at the time of HTx [2, 3]. Causes for increased perioperative mortality are more complex surgical procedure, often requiring major reconstructive surgery and a higher bleeding risk due to previous sternotomies. Donor right ventricular failure secondary to pulmonary hypertension may contribute as well. The highest risk for perioperative death was documented in neonates requiring circulatory support with ECMO prior to transplantation [15]. Patients with CHD also have an increased risk of rejection due to younger age, a more powerful immune system and increased levels of preformed HLA antibodies from prior blood transfusions and homograft implants at the time of reparative cardiac surgeries. Particularly the need for reconstruction of pulmonary arteries has been identified as a risk factor for increased perioperative and long-term mortality in some series [16]. Single ventricles following palliation have the worst outcome with 1, 5, and 10-year survival of 70%, 58%, and 50% [50].

In Fontan patients <18 years of age when listed, Bernstein et al. [51] reported the outcome of HTx. Survival was 77% at 1 year and 67% at 5 years, significantly lower compared with 91% 1-year and 81% 5-year HTx survival for noncongenital diagnosis. Overall mortality for HTx after Fontan failure was 33%. This data suggests that the increased risk in Fontan patients is predominantly in the early period after transplantation. In the Fontan group, there was a high rate of early graft failure (17%), infections (30%), and hemorrhage/operative complications (9%) as a cause of death, compared with other CHD and non CHD diagnosis [52]. In a single-institution experience with 155 patients undergoing HTX for CHD, 43 had a previous Fontan procedure: in 39.5% they had protein-losing enteropathy, 41.8% chronic heart failure, and 9.3% acute post-Fontan failure [39]. Patients undergoing HTx due to “failing” Fontan were more likely to require pulmonary artery reconstruction, which was necessary in 85.4% (*P* < 0.0001) and had longer cardiopulmonary bypass times (*P* < 0.0001). The 90-day mortality rate was 35% versus 20% in other CHD (*P* = 0.055). Average age in this patient group was 14.5 years [1–12, 14–48]. Only 12 patients were at least 20 years old at the time of HTx, and only 3 patients older than 30 years. Of these 12 patients, 7 died during followup. 90-day mortality did not differ between the age groups; however, after 1999 90 day mortality was 14.3% in those <18 years and 50% in those at least 18 years old (*P* = 0.09). So in patients >18 years old with failed Fontan the outcomes after HTx are still not well defined. In selected patients conversion of an atriopulmonary Fontan to an extracardiac total cavopulmonary anastomosis with arrhythmia surgery may even be the preferred treatment strategy to postpone the need for transplantation.

Overall, outcome for HTx for complex CHD has improved annually over the past 20 years [16]; the highest risk was transplant prior to 1990 (*P* = 0.01), respectively prior to 1996 (*P* = 0.001). Males also had a better outcome (*P* = 0.02) [16]. Neonates had a worse outcome (*P* = 0.005). Still, compared to patients with dilated cardiomyopathy in most studies mortality in patients with CHD after HTx was slightly worse than after dilated cardiomyopathy. In our own experience, long-term survival after heart transplantation performed in 13 adolescents and adults with CHD (mean age of 27.5 years) was 77% 20 years, not significantly different from an age-matched control-group transplanted for dilated cardiomyopathy [53]. In a recent report pediatric patients with CHD were found to have a trend towards an increased risk of cardiac allograft vasculopathy with a relative risk of 1.36 (*P* = 0.08) potentially due to increased preformed antibodies [15].

## 13. Heart-Lung or Lung Transplantation

Combined heart and lung transplantation or lung transplantation with intracardiac repair of the CHD is considered for patients with irreversible pulmonary hypertension or inadequate sized pulmonary arteries (complex forms of pulmonary atresia). The first heart-lung transplantation was performed in 1981 in a patient with pulmonary arterial hypertension [54]. The highest number of procedures was performed in 1989 with a total of 284 in adult and 61 in pediatric patients [55]. More recently, the numbers have been decreasing again. In adult patients, the main cause for heart-lung transplantation is Eisenmenger's syndrome [56] followed by idiopathic pulmonary arterial hypertension and cystic fibrosis.

Double-lung transplantation with intracardiac repair of the cardiac defect is an option in those with irreversible pulmonary hypertension and simple intracardiac defects, such as atrial or ventricular septal defects, aortopulmonary windows, or Eisenmenger syndrome due to a large patent ductus arteriosus [38].

In a series of including patients with adult CHD (ventricular and atrial septal defect, patent ductus arteriosus, etc.), 46 underwent heart-lung transplantations and 5 had double lung transplantations [57]. In this series, survival was 80%, 69%, and 53% at 1 year, 5 years, and 10 years: this was not different from survival in patients without CHD. So, despite high operative mortality due to a higher incidence of complications, long-term prognosis in adult CHD undergoing lung transplantation is excellent.

## 14. Conclusion

There is a growing cohort of young adults with complex congenital heart disease, many at risk for premature death due to heart failure. Heart transplantation (HTx) is and will be an important treatment option for selected ACHD patients with end-stage circulatory failure. It improves quality of life and survival in selected patients. Obstacles for successful transplantation are technical challenges due to complex anatomy and previous operations. In the absence of validated tools for prognostication, optimal timing for transplantation listing remains a major challenge and requires close multidisciplinary collaboration between ACHD specialists, heart failure specialists, surgeons and many others. Institutional protocols for serial followup of potential candidates are essential. Mechanical circulatory support as a bridge to HTx is an important option to consider especially in children waiting for HTx.

## Figures and Tables

**Table 1:** Main reasons for heart transplantation in congenital heart disease.

(i) Primary heart transplantation (newborn HTx for HLHS or Ebstein's anomaly)	
(ii) Eisenmenger syndrome with simple CHD with left to right shunting (acyanotic)	
(iii) Tetralogy of Fallot (repaired or palliated), and other complex CHD (cyanotic)	
(iv) Univentricular heart with and without Fontan circulation	
(v) Systemic right ventricle (CCTGA or TGA after atrial switch operation)	

HTx: heart transplantation; HLHS: hypoplastic left heart syndrome; CHD: congenital heart disease; CCTGA: congenitally corrected transposition of the great arteries; TGA: transposition of the great arteries.

**Table 2:** Etiology of heart failure in congenital heart disease.

Primary heart dysfunction	
Valvular regurgitation or stenosis	
Congenital shunt lesions or systemic pulmonary shunts	
Long-standing cyanosis	
Arrhythmias	
Dysfunction of the subpulmonic ventricle	
Inherited abnormalities of the myocardium	
Oxygen supply-demand mismatch	
The Fontan circulation per se	
Associated congenital heart disease in visceral heterotaxy	

**Table 3:** Contraindications for heart transplantation in adult congenital heart disease.

Absolute contraindications	
Active infections (active hepatitis C and viral replication)	
Severe metabolic disease	
Other multiple severe congenital anomalies	
Multisystem organ failure	
Active malignancy	
Cognitive/behavioral disability that interferes with compliance	

Relative contraindications with increased morbidity	
Diabetes with end-organ dysfunction	
Irreversible renal dysfunction	
Symptomatic cerebrovascular or peripheral vascular disease not amenable to revascularization	
Obesity with a pretransplant BMI >30 kg/m^2^	
Active tobacco smoking and substance abuse	
Elevated PVR	
HIV positivity	
Asplenia in right atrial isomerism	
Residual shunts (high-output heart failure post-transplant)	

BMI: body mass index; PVR: pulmonary vascular resistance; HIV: human immunodeficiency virus.

**Table 4:** Risk factors for heart transplantation according to Lamour et al. 2009 [13].

Variable	Relative risk	*P* value
Early phase		
Previous Fontan	8.6	0.003
Higher pretransplant mean RAP (only if previous Fontan)	2.4	<0.0001
Longer ischemic time	1.6	0.002
Older recipient age	1.5	0.02
Interaction of donor age and ischemic time	1.4	0.0007
Constant phase		
Previous classic Glenn operation	3.1	0.01
CMV +donor, CMV −recipient	2.8	0.001
Higher systolic transpulmonary gradient	2.0	0.01
Younger recipient age	1.8	0.0001
Univentricular heart, previous cardiac surgery, >1 year old	6.48	<0.0001

RAP: right atrial pressure; CMV: cytomegalovirus.
